# Supplementary material for: A Likelihood Approach for Real-Time Calibration of Stochastic Compartmental Epidemic Models
Source: PLoS Comput Biol. 2017 Jan 17;13(1):e1005257. doi: 10.1371/journal.pcbi.1005257 (PMC5240920; doi:10.1371/journal.pcbi.1005257)
Supplement: S1 File — (TAR.GZ) [file pcbi.1005257.s014.tar.gz › HSPH_Online-SI-Revision/IPoi09/n100-mild/n100-mild_BENCH2_table-4.pdf]

1	newly 3 weeks spec	3503.	5008.63	{3174., 7534.}	55.121	0.218523	newly 3 weeks cumul	8908.	16 604.8	{7802., 20 653.}	95.9853	0.324106	newly 5 weeks spec	3621.	3209.15	{1394., 6675.}	48.3374	0.258526	newly 5 weeks cumul	16 248.	23 937.4	{17 998., 32 063.}	57.1808	0.220391	newly 56 weeks cumul	29 432.	32 591.	{20 856., 46 952.}	29.5816	0.122394
2	newly 3 weeks spec	4175.	4257.96	{2178., 6974.}	39.0135	0.196647	newly 3 weeks cumul	10 673.	15 175.2	{10 120., 21 287.}	53.3766	0.213002	newly 5 weeks spec	4247.	2458.	{831., 6192.}	55.7335	0.392949	newly 5 weeks cumul	19 311.	20 950.8	{12 308., 30 619.}	32.948	0.15627	newly 55 weeks cumul	34 724.	27 348.4	{14 978., 44 146.}	33.808	0.193203
3	newly 3 weeks spec	4167.	4674.75	{1521., 7735.}	45.2976	0.196148	newly 3 weeks cumul	10 718.	17 635.2	{8758., 22 880.}	68.5552	0.215735	newly 5 weeks spec	4114.	2011.2	{409., 4240.}	54.8371	0.438887	newly 5 weeks cumul	19 213.	22 853.4	{10 050., 32 665.}	38.2267	0.151921	newly 58 weeks cumul	31 221.	25 597.8	{10 601., 38 584.}	29.3258	0.166326
4	newly 3 weeks spec	8072.	8816.88	{4717., 13 686.}	39.7792	0.170692	newly 3 weeks cumul	18 303.	31 603.7	{24 744., 37 824.}	75.5483	0.245006	newly 5 weeks spec	6729.	2981.73	{837., 5955.}	63.0529	0.511168	newly 5 weeks cumul	33 292.	40 083.	{29 627., 49 852.}	26.415	0.100451	newly 62 weeks cumul	45 410.	42 880.8	{30 084., 55 700.}	18.45	0.0876852
5	newly 3 weeks spec	3934.	6455.69	{3467., 9419.}	70.1263	0.218672	newly 3 weeks cumul	10 428.	22 839.3	{17 061., 27 057.}	119.019	0.332955	newly 5 weeks spec	3936.	3184.81	{1061., 8100.}	50.1534	0.275125	newly 5 weeks cumul	18 434.	30 744.1	{20 076., 38 104.}	67.789	0.214549	newly 58 weeks cumul	32 210.	35 386.8	{21 142., 52 123.}	26.4512	0.109971
6	newly 3 weeks spec	4474.	5204.59	{2641., 8427.}	46.9998	0.195204	newly 3 weeks cumul	11 885.	18 127.	{8896., 24 260.}	62.4649	0.211026	newly 5 weeks spec	4394.	2924.27	{992., 6455.}	54.0158	0.369852	newly 5 weeks cumul	20 970.	25 056.	{15 228., 36 347.}	37.9826	0.150441	newly 56 weeks cumul	33 539.	30 465.5	{16 754., 46 311.}	32.5098	0.169387
7	newly 3 weeks spec	5537.	7136.32	{3593., 9978.}	47.4436	0.21762	newly 3 weeks cumul	11 792.	22 223.9	{16 577., 27 215.}	97.2743	0.34506	newly 5 weeks spec	6137.	3512.5	{934., 6618.}	49.6252	0.37889	newly 5 weeks cumul	24 548.	31 210.5	{21 693., 37 975.}	38.9467	0.184245	newly 61 weeks cumul	35 993.	37 327.4	{26 865., 46 342.}	20.2292	0.0893236
8	newly 3 weeks spec	3810.	7491.03	{5584., 9109.}	98.1151	0.289662	newly 3 weeks cumul	10 632.	22 249.6	{13 218., 26 062.}	112.081	0.320302	newly 5 weeks spec	3274.	5692.21	{2964., 8932.}	78.4844	0.234491	newly 5 weeks cumul	17 804.	34 681.7	{28 693., 39 726.}	95.5694	0.286259	newly 55 weeks cumul	26 742.	50 128.2	{35 174., 63 929.}	87.7641	0.26454
9	newly 3 weeks spec	6820.	9004.35	{5819., 12 007.}	40.5	0.149772	newly 3 weeks cumul	16 298.	28 763.2	{24 467., 32 646.}	81.0992	0.265416	newly 5 weeks spec	6191.	4411.19	{2168., 8332.}	44.6246	0.268017	newly 5 weeks cumul	29 431.	39 884.9	{31 542., 46 248.}	38.1301	0.138868	newly 60 weeks cumul	43 358.	46 169.2	{37 910., 56 900.}	16.0107	0.0669287
10	newly 3 weeks spec	3895.	2885.46	{740., 5813.}	47.23	0.305028	newly 3 weeks cumul	10 575.	11 307.9	{3940., 19 213.}	44.2023	0.201849	newly 5 weeks spec	3765.	1483.36	{287., 3546.}	65.868	0.593782	newly 5 weeks cumul	18 373.	14 898.7	{4758., 27 650.}	41.2284	0.244923	newly 54 weeks cumul	31 484.	17 655.8	{5142., 33 775.}	48.5075	0.356596
11	newly 3 weeks spec	5349.	8960.62	{6117., 10 399.}	71.4042	0.232065	newly 3 weeks cumul	11 903.	22 390.9	{11 660., 29 702.}	94.1385	0.280697	newly 5 weeks spec	6127.	8359.15	{5768., 10 273.}	39.7978	0.140418	newly 5 weeks cumul	24 206.	39 927.	{30 387., 44 816.}	66.5259	0.218306	newly 58 weeks cumul	41 009.	62 467.9	{53 554., 68 433.}	52.3274	0.181038
12	newly 3 weeks spec	4227.	6729.47	{4796., 8463.}	60.4659	0.196681	newly 3 weeks cumul	11 278.	21 576.4	{16 834., 25 018.}	91.3145	0.276394	newly 5 weeks spec	4123.	4671.95	{2163., 8639.}	44.7298	0.177262	newly 5 weeks cumul	19 649.	32 006.4	{24 831., 37 752.}	63.056	0.206313	newly 54 weeks cumul	37 494.	43 696.8	{29 255., 62 525.}	28.3719	0.108761
13	newly 3 weeks spec	5374.	8461.25	{5057., 10 689.}	59.3805	0.190988	newly 3 weeks cumul	12 699.	25 814.2	{22 063., 28 875.}	103.278	0.305273	newly 5 weeks spec	5146.	4738.86	{1643., 9335.}	40.8722	0.213066	newly 5 weeks cumul	23 362.	37 264.3	{27 727., 42 533.}	59.5083	0.197435	newly 59 weeks cumul	36 672.	44 398.4	{30 126., 55 459.}	28.272	0.106524
14	newly 3 weeks spec	7541.	8269.73	{3285., 12 616.}	39.6258	0.179239	newly 3 weeks cumul	19 010.	27 442.6	{17 657., 34 250.}	52.7695	0.192721	newly 5 weeks spec	5669.	4008.36	{836., 10 649.}	60.4708	0.406383	newly 5 weeks cumul	32 236.	37 504.7	{23 040., 46 303.}	28.6042	0.115967	newly 61 weeks cumul	43 111.	42 551.6	{25 626., 55 770.}	21.2035	0.100397
15	newly 3 weeks spec	6533.	8489.56	{3552., 11 851.}	51.3916	0.215393	newly 3 weeks cumul	14 286.	19 095.1	{6238., 31 370.}	61.5426	0.252946	newly 5 weeks spec	6860.	10 312.5	{7737., 11 878.}	55.1625	0.192896	newly 5 weeks cumul	28 288.	39 302.2	{21 964., 51 726.}	51.8971	0.196301	newly 58 weeks cumul	44 155.	85 620.4	{80 235., 90 843.}	93.9086	0.286882
16	newly 3 weeks spec	2942.	5420.67	{3054., 7384.}	89.9237	0.267427	newly 3 weeks cumul	7823.	17 804.5	{12 917., 21 441.}	127.591	0.345335	newly 5 weeks spec	2786.	3406.56	{1334., 5837.}	55.4585	0.2191	newly 5 weeks cumul	13 642.	25 630.4	{16 311., 32 989.}	91.3611	0.27233	newly 54 weeks cumul	27 181.	33 109.1	{18 793., 47 720.}	37.4528	0.145565
17	newly 3 weeks spec	6206.	7040.04	{4486., 9982.}	33.1944	0.148838	newly 3 weeks cumul	13 556.	24 054.3	{19 821., 28 379.}	85.2717	0.296374	newly 5 weeks spec	6327.	3339.17	{1453., 5661.}	52.846	0.372661	newly 5 weeks cumul	26 549.	32 496.	{25 800., 40 327.}	30.3737	0.126734	newly 60 weeks cumul	43 173.	38 312.5	{28 384., 49 055.}	19.6067	0.0963622
18	newly 3 weeks spec	7370.	14 392.2	{10 543., 16 589.}	95.2803	0.284653	newly 3 weeks cumul	13 588.	34 671.7	{24 733., 41 026.}	155.164	0.398024	newly 5 weeks spec	8646.	5631.58	{1929., 10 092.}	42.3888	0.2746	newly 5 weeks cumul	31 571.	50 929.8	{48 133., 52 895.}	61.3182	0.207189	newly 64 weeks cumul	43 150.	55 027.9	{49 332., 58 162.}	27.5269	0.104657
19	newly 3 weeks spec	4209.	6067.04	{4371., 7489.}	49.637	0.174279	newly 3 weeks cumul	10 513.	18 362.5	{11 600., 21 329.}	74.6649	0.234045	newly 5 weeks spec	4445.	4419.33	{2029., 7113.}	38.956	0.188187	newly 5 weeks cumul	19 470.	28 202.5	{21 514., 33 156.}	48.4198	0.170041	newly 55 weeks cumul	33 382.	39 087.8	{25 361., 51 518.}	29.0255	0.116402
20	newly 3 weeks spec	5435.	10 942.6	{6059., 13 768.}	108.694	0.3239	newly 3 weeks cumul	9479.	24 839.	{9188., 36 109.}	171.387	0.422033	newly 5 weeks spec	7800.	7472.68	{3448., 13 549.}	42.9923	0.203457	newly 5 weeks cumul	25 122.	42 340.9	{34 556., 46 962.}	71.9116	0.236265	newly 64 weeks cumul	39 842.	51 199.	{48 378., 53 875.}	28.5051	0.10853
21	newly 3 weeks spec	6939.	6390.94	{2141., 12 592.}	41.3469	0.228309	newly 3 weeks cumul	13 970.	22 960.5	{12 886., 28 634.}	67.3488	0.21408	newly 5 weeks spec	7245.	1988.75	{102., 7602.}	76.5528	0.9028	newly 5 weeks cumul	29 481.	29 075.2	{20 613., 36 133.}	17.1256	0.0815862	newly 64 weeks cumul	42 520.	30 151.5	{20 693., 39 332.}	29.0887	0.16425
22	newly 3 weeks spec	3601.	3782.43	{1207., 6752.}	46.2034	0.216087	newly 3 weeks cumul	9861.	13 763.6	{6111., 20 205.}	52.9398	0.184622	newly 5 weeks spec	3826.	2107.43	{449., 4794.}	56.0096	0.433178	newly 5 weeks cumul	17 518.	18 756.5	{7359., 29 943.}	39.1328	0.176536	newly 54 weeks cumul	36 374.	22 997.	{8056., 39 506.}	40.8514	0.276488
23	newly 3 weeks spec	5953.	7401.76	{4441., 9329.}	35.5928	0.14038	newly 3 weeks cumul	13 022.	21 065.	{11 422., 26 721.}	71.7303	0.244439	newly 5 weeks spec	6752.	5313.64	{2668., 8494.}	33.7684	0.202324	newly 5 weeks cumul	26 990.	32 938.1	{24 498., 37 628.}	27.8118	0.108756	newly 59 weeks cumul	42 711.	43 716.3	{33 489., 52 028.}	15.8848	0.0700921
24	newly 3 weeks spec	7664.	10 029.3	{7275., 12 752.}	37.8685	0.138393	newly 3 weeks cumul	15 273.	29 873.3	{20 209., 35 186.}	100.69	0.303856	newly 5 weeks spec	6606.	4539.44	{1416., 9965.}	56.3112	0.36601	newly 5 weeks cumul	30 012.	41 885.4	{37 975., 44 935.}	40.1454	0.144935	newly 63 weeks cumul	39 935.	46 204.	{39 536., 53 809.}	16.9481	0.0661078
25	newly 3 weeks spec	4080.	5198.52	{3412., 7012.}	38.2715	0.142228	newly 3 weeks cumul	9690.	16 430.	{9118., 19 444.}	74.4968	0.237689	newly 5 weeks spec	4627.	3603.7	{1639., 5659.}	35.4159	0.209972	newly 5 weeks cumul	18 986.	24 463.	{17 596., 30 245.}	34.7785	0.128094	newly 54 weeks cumul	37 102.	33 769.4	{20 580., 44 158.}	22.8931	0.115997
26	newly 3 weeks spec	5222.	5144.89	{3174., 7534.}	27.6409	0.162844	newly 3 weeks cumul	12 723.	16 837.	{7802., 20 960.}	46.3931	0.212541	newly 5 weeks spec	5352.	3317.56	{1394., 6675.}	45.1683	0.315793	newly 5 weeks cumul	23 692.	24 412.5	{17 998., 32 063.}	22.2382	0.126291	newly 56 weeks cumul	38 625.	33 268.1	{21 768., 46 952.}	24.946	0.131904
27	newly 3 weeks spec	4332.	7283.05	{5637., 8281.}	72.1139	0.2366	newly 3 weeks cumul	10 828.	20 151.7	{12 410., 24 515.}	92.3309	0.282987	newly 5 weeks spec	4581.	6425.88	{4854., 8120.}	42.1018	0.145675	newly 5 weeks cumul	20 103.	33 643.1	{27 669., 37 298.}	69.8606	0.229073	newly 54 weeks cumul	36 608.	56 100.	{47 735., 65 979.}	53.2451	0.181739
28	newly 3 weeks spec	3391.	4640.	{2003., 7027.}	52.7573	0.186729	newly 3 weeks cumul	9081.	15 637.	{7966., 20 133.}	76.3037	0.236365	newly 5 weeks spec	3182.	2885.46	{740., 5813.}	50.4254	0.276664	newly 5 weeks cumul	15 833.	22 304.9	{11 094., 30 492.}	50.9602	0.177264	newly 55 weeks cumul	31 926.	28 652.8	{12 299., 43 354.}	31.0453	0.164306
29	newly 3 weeks spec	3449.	4780.05	{2342., 7020.}	52.7744	0.183076	newly 3 weeks cumul	8407.	15 798.8	{11 509., 20 121.}	90.0565	0.270062	newly 5 weeks spec	3362.	2793.9	{763., 5537.}	51.2422	0.30426	newly 5 weeks cumul	15 415.	22 411.2	{13 548., 29 249.}	50.0984	0.168478	newly 57 weeks cumul	26 694.	27 432.	{14 746., 39 315.}	29.7208	0.134408
30	newly 3 weeks spec	6917.	8206.57	{1977., 10 775.}	44.1411	0.240869	newly 3 weeks cumul	15 845.	22 677.3	{3126., 30 122.}	71.0905	0.337725	newly 5 weeks spec	6557.	6044.65	{3061., 9308.}	28.5599	0.156649	newly 5 weeks cumul	29 817.	36 103.	{13 768., 44 541.}	40.4808	0.198531	newly 59 weeks cumul	44 183.	52 997.	{43 383., 62 182.}	22.3427	0.0859963
31	newly 3 weeks spec	4050.	7148.33	{3937., 9025.}	85.6061	0.294191	newly 3 weeks cumul	9667.	19 799.9	{8536., 25 896.}	115.985	0.358455	newly 5 weeks spec	4228.	6212.17	{2744., 8596.}	61.9391	0.231057	newly 5 weeks cumul	18 314.	32 962.6	{21 697., 39 806.}	86.8572	0.290453	newly 56 weeks cumul	33 046.	53 427.	{39 461., 64 979.}	65.1627	0.214228
32	newly 3 weeks spec	3828.	3782.43	{1207., 6752.}	43.8871	0.217983	newly 3 weeks cumul	10 480.	13 763.6	{6111., 20 205.}	46.9667	0.171856	newly 5 weeks spec	3614.	2107.43	{449., 4794.}	55.6586	0.417841	newly 5 weeks cumul	17 929.	18 756.5	{7359., 29 943.}	38.1417	0.17612	newly 54 weeks cumul	35 777.	22 997.	{8056., 39 506.}	40.5795	0.272381
33	newly 3 weeks spec	6228.	9780.15	{1640., 12 793.}	83.4494	0.339643	newly 3 weeks cumul	10 354.	20 281.5	{2685., 31 396.}	123.296	0.420984	newly 5 weeks spec	7272.	7387.12	{3613., 10 705.}	27.5763	0.142679	newly 5 weeks cumul	26 028.	37 218.8	{16 576., 44 909.}	58.3347	0.238029	newly 64 weeks cumul	32 752.	49 656.2	{47 811., 51 619.}	51.6127	0.180174
34	newly 3 weeks spec	7515.	12 009.1	{9367., 13 443.}	59.8021	0.199197	newly 3 weeks cumul	17 041.	33 579.1	{28 708., 36 376.}	98.013	0.294184	newly 5 weeks spec	6043.	6739.1	{3667., 9391.}	30.5585	0.124527	newly 5 weeks cumul	30 687.	50 037.3	{44 723., 52 852.}	63.0568	0.211243	newly 60 weeks cumul	40 483.	59 862.7	{49 632., 65 859.}	47.8712	0.167677
35	newly 3 weeks spec	11 144.	5689.26	{1524., 8603.}	48.9478	0.563416	newly 3 weeks cumul	14 898.	11 895.2	{2264., 23 357.}	44.5662	0.505955	newly 5 weeks spec	7436.	6068.61	{2165., 8620.}	24.8439	0.341035	newly 5 weeks cumul	37 536.	24 403.9	{12 027., 32 710.}	34.9853	0.444558	newly 64 weeks cumul	40 652.	36 362.3	{34 665., 39 437.}	10.59	0.27223
36	newly 3 weeks spec	4855.	14 392.2	{10 543., 16 589.}	196.44	0.465931	newly 3 weeks cumul	9201.	34 671.7	{24 733., 41 026.}	276.825	0.567344	newly 5 weeks spec	5772.	5631.58	{1929., 10 092.}	41.2252	0.198229	newly 5 weeks cumul	20 947.	50 929.8	{48 133., 52 895.}	143.136	0.385356	newly 64 weeks cumul	32 008.	55 027.9	{49 332., 58 162.}	71.9191	0.234379
37	newly 3 weeks spec	5673.	7558.82	{4933., 9451.}	38.4774	0.138146	newly 3 weeks cumul	13 815.	21 544.9	{15 006., 26 566.}	58.0883	0.191559	newly 5 weeks spec	5464.	5739.	{2022., 9231.}	42.0179	0.199096	newly 5 weeks cumul	25 233.	34 195.1	{25 866., 39 673.}	36.231	0.12987	newly 57 weeks cumul	39 323.	46 608.3	{28 987., 58 427.}	29.8845	0.118325
38	newly 3 weeks spec	6180.	10 942.6	{6059., 13 768.}	85.6214	0.277724	newly 3 weeks cumul	10 916.	24 839.	{9188., 36 109.}	139.149	0.3769	newly 5 weeks spec	7800.	7472.68	{3448., 13 549.}	42.9923	0.203457	newly 5 weeks cumul	26 749.	42 340.9	{34 556., 46 962.}	61.9418	0.211192	newly 64 weeks cumul	37 595.	51 199.	{48 378., 53 875.}	36.1857	0.133741
39	newly 3 weeks spec	5821.	8269.73	{3285., 12 616.}	60.9319	0.216872	newly 3 weeks cumul	14 912.	27 442.6	{17 657., 34 250.}	89.5969	0.278917	newly 5 weeks spec	4560.	4008.36	{836., 10 649.}	62.9226	0.359258	newly 5 weeks cumul	25 319.	37 504.7	{23 040., 46 303.}	52.6062	0.178311	newly 61 weeks cumul	34 866.	42 551.6	{25 626., 55 770.}	33.2601	0.128465
40	newly 3 weeks spec	8986.	10 029.3	{7275., 12 752.}	25.9809	0.108559	newly 3 weeks cumul	18 610.	29 873.3	{20 209., 35 186.}	66.9244	0.228491	newly 5 weeks spec	6981.	4539.44	{1416., 9965.}	55.6499	0.376561	newly 5 weeks cumul	34 664.	41 885.4	{37 975., 44 935.}	22.5692	0.0880516	newly 63 weeks cumul	43 420.	46 204.	{39 536., 53 809.}	11.4794	0.0474499
41	newly 3 weeks spec	8035.	11 325.9	{6897., 14 949.}	46.5703	0.161993	newly 3 weeks cumul	14 755.	32 954.9	{20 465., 37 308.}	123.536	0.338595	newly 5 weeks spec	8819.	3458.37	{893., 8394.}	65.2392	0.545657	newly 5 weeks cumul	33 711.	43 520.6	{38 170., 47 106.}	29.0992	0.109861	newly 64 weeks cumul	47 457.	45 675.8	{38 873., 50 744.}	6.72047	0.0308448
42	newly 3 weeks spec	6821.	10 234.4	{4287., 12 870.}	61.5704	0.220897	newly 3 weeks cumul	14 678.	28 055.6	{6835., 35 533.}	105.55	0.331237	newly 5 weeks spec	6589.	6781.23	{3196., 12 512.}	38.9986	0.175048	newly 5 weeks cumul	28 856.	43 604.6	{27 301., 49 711.}	55.7366	0.193563	newly 61 weeks cumul	41 167.	55 687.9	{47 010., 64 696.}	36.0788	0.131303
43	newly 3 weeks spec	6531.	14 242.5	{10 346., 16 589.}	118.076	0.332332	newly 3 weeks cumul	12 527.	35 221.7	{27 570., 41 026.}	181.166	0.442509	newly 5 weeks spec	8332.	5147.64	{1849., 8969.}	43.9205	0.29079	newly 5 weeks cumul	29 200.	50 424.8	{45 244., 52 895.}	72.6877	0.236488	newly 64 weeks cumul	45 957.	54 076.7	{46 766., 58 104.}	17.9448	0.0704774
44	newly 3 weeks spec	8080.	12 214.7	{10 158., 14 645.}	58.56	0.210981	newly 3 weeks cumul	15 523.	29 444.4	{17 524., 38 331.}	98.6405	0.302901	newly 5 weeks spec	8641.	7421.16	{3379., 12 375.}	39.6732	0.211716	newly 5 weeks cumul	34 157.	47 485.5	{43 728., 51 448.}	42.3618	0.155647	newly 63 weeks cumul	46 278.	56 380.5	{50 906., 60 202.}	21.8299	0.0848964
45	newly 3 weeks spec	4973.	4858.88	{2108., 8833.}	46.9503	0.218507	newly 3 weeks cumul	11 874.	20 026.	{12 994., 27 671.}	74.7364	0.242585	newly 5 weeks spec	4999.	1953.	{620., 4778.}	64.8786	0.553707	newly 5 weeks cumul	22 191.	25 129.2	{14 901., 38 416.}	33.707	0.132233	newly 59 weeks cumul	37 756.	28 588.9	{15 664., 44 701.}	33.1201	0.186892
46	newly 3 weeks spec	6137.	8399.5	{4690., 10 093.}	48.2728	0.189472	newly 3 weeks cumul	13 041.	23 319.2	{12 523., 28 239.}	89.8445	0.297913	newly 5 weeks spec	7235.	5542.96	{3304., 8858.}	32.0063	0.181751	newly 5 weeks cumul	27 607.	36 112.8	{25 980., 40 327.}	38.3122	0.149943	newly 60 weeks cumul	46 416.	47 175.4	{40 553., 53 847.}	10.163	0.0450869
47	newly 3 weeks spec	7497.	8211.42	{5457., 10 131.}	21.1608	0.087797	newly 3 weeks cumul	15 877.	25 904.3	{17 385., 29 558.}	64.5665	0.211454	newly 5 weeks spec	7293.	4364.08	{1867., 9613.}	48.9859	0.320289	newly 5 weeks cumul	31 506.	36 584.7	{29 482., 40 740.}	18.2515	0.0718004	newly 60 weeks cumul	45 078.	42 949.7	{31 470., 54 332.}	13.385	0.0623964
48	newly 3 weeks spec	5435.	6884.47	{2426., 8970.}	46.347	0.190942	newly 3 weeks cumul	12 559.	17 116.	{4900., 25 317.}	61.1422	0.239826	newly 5 weeks spec	5927.	7554.36	{5835., 8772.}	31.6704	0.120073	newly 5 weeks cumul	24 449.	32 160.4	{15 036., 40 030.}	44.8468	0.173324	newly 55 weeks cumul	39 759.	67 607.4	{56 799., 75 169.}	70.0431	0.228749
49	newly 3 weeks spec	5594.	4857.45	{1368., 8361.}	37.8457	0.217223	newly 3 weeks cumul	12 132.	17 934.7	{9468., 22 637.}	60.0988	0.212966	newly 5 weeks spec	4978.	1958.18	{210., 4636.}	64.8745	0.618079	newly 5 weeks cumul	22 959.	23 111.	{10 687., 31 103.}	23.9179	0.116266	newly 61 weeks cumul	32 589.	25 579.3	{10 821., 36 060.}	26.512	0.156284
50	newly 3 weeks spec	6652.	9774.06	{5291., 12 859.}	59.6531	0.216919	newly 3 weeks cumul	14 670.	26 286.4	{8940., 34 316.}	95.7753	0.321171	newly 5 weeks spec	6004.	6053.67	{1680., 12 054.}	50.7111	0.25138	newly 5 weeks cumul	27 698.	40 609.8	{31 743., 46 603.}	51.3541	0.18485	newly 62 weeks cumul	37 321.	49 665.1	{38 169., 56 440.}	33.9093	0.123572
